# Laughter in everyday life: an event-based experience sampling method study using wrist-worn wearables

**DOI:** 10.3389/fpsyg.2024.1296955

**Published:** 2024-05-02

**Authors:** Stefan Stieger, Selina Volsa, David Willinger, David Lewetz, Bernad Batinic

**Affiliations:** ^1^Department of Psychology and Psychodynamics, Karl Landsteiner University of Health Sciences, Krems an der Donau, Austria; ^2^Department of Work, Organizational, and Media Psychology, Johannes Kepler University, Linz, Austria

**Keywords:** laughing, fit of laughter, experience sampling, wearable, physical analogue scale, gender, personality, gelotophobia

## Abstract

Laughter is a universal, nonverbal vocal expression of broad significance for humans. Interestingly, rather little is known about how often we laugh and how laughter is associated with our personality. In a large, event-based, experience sampling method study (*N* = 52; *k* = 9,261 assessments) using wrist-worn wearables and a physical analogue scale, we analyzed belly laughs and fit of laughter events in participants’ everyday life for 4 weeks. Additionally, we assessed associations with laughter frequency such as personality, happiness, life satisfaction, gelotophobia (i.e., fear of being laughed at), and cheerfulness. Validating our new measurement approach (i.e., wearables, physical analogue scale), laughter events elicited higher happiness ratings compared to reference assessments, as expected. On average, participants reported 2.5 belly laughs per day and on every fourth day a fit of laughter. As expected, participants who were happier and more satisfied with their life laughed more frequently than unhappier, unsatisfied participants. Women and younger participants laughed significantly more than men and older participants. Regarding personality, laughter frequency was positively associated with openness and conscientiousness. No significant association was found for gelotophobia, and results for cheerfulness and related concepts were mixed. By using state-of-the-art statistical methods (i.e., recurrent event regression) for the event-based, multi-level data on laughter, we could replicate past results on laughing.

## 1 Introduction

Laughter is a universal, nonverbal vocal expression, which usually communicates cooperation and positive affect between humans (Bryant et al., 2016). Despite its broad significance for humans, comparatively little is known about laughter type (e.g., belly laugh, fit of laughter) and laughter frequency in association with our psyche, such as personality, well-being, but also more specific ones, such as cheerfulness or gelotophobia. One reason for this might be the difficulty of assessing laughter events in everyday life, because laughter is a rather fleeting expression at the threshold of perception, which can hardly be remembered using cross-sectional or even daily diary designs (early diary studies, e.g., Graeven and Morris, 1975). Therefore, recent research has often focused on experience sampling method designs (ESM; Mannell and McMahon, 1982; Larson and Csikszentmihalyi, 1983; Mehl and Conner, 2011; Bolger and Laurenceau, 2013; Trull and Ebner-Priemer, 2013; Csikszentmihalyi and Larson, 2014) which, although more difficult to implement, allow assessment of the laughter event at the time when it happens by using printed diaries or, more recently, electronic devices such as smartphones or wearables.

Using an event-contingent ESM procedure, Mannell and McMahon (1982) conducted one of the first studies on the topic of laughter in everyday life. They had 31 students record the occurrence of all their humorous experiences during the day, but did not explicitly ask for laughter incidences. In two further seminal papers (Kuiper and Martin, 1998; Martin and Kuiper, 1999), the frequency of laughter was measured using paper-and-pencil diaries, which assessed several variables in a daily laughter record (DLR; e.g., source of humor, strength of mirth, initiator, and time of the day). The authors found that laughing (defined as spontaneous laughter, not smiling or any other forms of laughter) occurred on average 18 times per day with a wide range of 0–89 laughs. In general, the frequency of laughter increased during the day, reaching its peak in the evening.

Despite the importance of laughter in everyday life, there is only a handful of other studies that attempted to replicate earlier studies but often used a different design. Vettin and Todt (2004) analyzed how often laughter occurs in natural conversations. Instead of using a classical diary or an ESM study using self-reports, they tape-recorded 48 h of conversations among dyads of friends and strangers in naturalistic settings (for a similar study also using tape-recordings, see Smoski and Bachorowski, 2003). They found that laughing was more frequent than previously reported in daily diary studies of laughter (Mannell and McMahon, 1982; Martin and Kuiper, 1999). They concluded, among other explanations, that people may not notice every instance of laughter. A further explanation is grounded in the procedure itself: some participants in paper-and-pencil diary or ESM studies may not always go to the trouble of retrieving their diaries to answer the questions, or may have been unable to record laughter incidences because the diary was forgotten or because they had no writing utensils. Another study by Valeri (2006) found laughter frequency patterns that were similar to those reported in Martin and Kuiper (1999), using a slightly modified version of the DLR; she reported a mean frequency of 19 laughs per day, again with a wide range (0–83 laughs).

In addition to these studies, there are further publications about laughter, most of which have revolved around other aspects (Reay, 2015: laughter in interview situations; Kashdan et al., 2014: laughter during social interactions) or used an observational design (Provine and Fischer, 1989; Provine, 1993). However, extant studies on the frequency of laughter in daily life are limited by the following points: first, most studies used a relatively small timespan of data collection (e.g., Kuiper and Martin, 1998; Martin and Kuiper, 1999: 3 days; Vettin and Todt, 2004: 2 days; Valeri, 2006: 1 day). This could reduce generalizability because short-term influences on laughter frequency (e.g., bad day, weekend, and holiday) can bias the results. Second, past research predominantly used paper-and-pencil diaries (e.g., Martin and Kuiper, 1999); this has the disadvantage that participant compliance with the intended procedure cannot be controlled (e.g., logging each laughter event when occurring). Some participants probably postpone the logging of laughter events (e.g., Stone, 2002) or even forget about them because it is too burdensome (e.g., finding the paper diary, searching for a pen, doing the assessment, and putting everything away again; Volsa et al., 2023). This problem is amplified by the fact that laughter in everyday life is a rather frequent behavior (e.g., Kuiper and Martin, 1998; up to 89 laughs per day). Furthermore, laughing often takes place in social situations where participants would find it awkward and impolite to interrupt the situation by filling in a questionnaire, i.e., the likelihood of postponing (and even forgetting) the assessment is heightened (because of their non-intrusive nature, wearables have the potential to alleviate this problem; see Volsa et al., 2023). Third, previous studies assessed potential correlates or moderators of the frequency of laughing events at the end of the day or study, rather than concurrently, i.e., longitudinally. This increases the risk of memory biases, because laughing is a human reaction taking place at the threshold of perception (i.e., can only reliably assessed in the situation when it occurs, but hardly remembered later).

The field of ESM methodology has experienced rapid growth in the past years, which is reflected in a rising number of publications pointing to the benefits of these designs (Gosling and Mason, 2015; Wrzus and Mehl, 2015; Davidson et al., 2017; Hamaker and Wichers, 2017). This growth is, in part, driven by the possibility of realizing ESM designs using smartphones and/or wearables (mobile ESM: e.g., Dufau et al., 2011; Miller, 2012; Harari et al., 2015, 2016). These devices greatly reduce the burden of implementing ESM designs because electronic questionnaires can be transmitted easily and the administration of the study can be easily achieved due to the fully-integrated, internet-based work-flow of the study itself (e.g., no printed paper-and-pencil questionnaires, central storage of data on the PI’s web-server; Lewetz and Stieger, 2023). In addition, these devices usually have many built-in sensors, which further augment their usefulness for the empirical sciences [e.g., GPS for mobility patterns; position sensors for the realization of measurement scales based on body-postures, e.g., physical analogue scale (PAS); Stieger et al., 2020]. All these new developments make it possible to analyze even subtle human behavior in the field (e.g., engagement with social media content; Stieger et al., 2022) that is short-lived (e.g., impact of watching funny YouTube videos; Stieger et al., 2023), and hardly remembered even minutes later (e.g., dreams; Stieger and Kuhlmann, 2018) when they have occurred in a manner that makes these data very valuable because of high data quality and accuracy.

Considering the discussion above, although laughter is a unique characteristic of human beings, there is currently very little research about the frequency of laughter in everyday life, as well as potential correlates of such (e.g., happiness, personality, satisfaction with life, gelotophobia, and cheerfulness; Martin and Kuiper, 1999; Vlahovic et al., 2012). In the present study, we present results from an ESM design on laughter by using the latest state-of-the-art methodology, i.e., event-based sampling procedure with one-button wearables and PAS (i.e., position of a participant’s forearm between flat and fully upright position as a response scale like a Visual Analogue Scale: Stieger et al., 2020; Volsa et al., 2023). The following explorative research questions were analyzed.

To validate our unique design, we first wanted to replicate a previous result that is related to laughter. Because one of the strongest associations with laughter is happiness (e.g., Vlahovic et al., 2012; explained variance up to 29%), we asked participants to judge their happiness after the laughing event by using the PAS [i.e., sensor-based gradual assessment of the angle position of the forearm from 0° (forearm laying on a flat surface) to 90° (forearm in a full upright position), similar to a visual analogue scale]. Happiness from the event-based sampling will be compared to happiness from time-based sampling (i.e., reference measurement of happiness in everyday life at random time points) by further accounting for other traits, such as gelotophobia, cheerfulness, and personality.

Research Question 1: Is happiness higher when laughing compared to when not laughing?

Furthermore, past research found several associations between laughter and other psychological concepts. For example, Martin and Kuiper (1999) found that men scoring high on a Type A personality (i.e., personalities that are more competitive, highly organized, ambitious, or aggressive are labeled Type A) laughed more than low-scoring Type A personality men. No association for women was found. In the present study, we want to focus on the more widely used Big Five personality concept. Because the strongest association between Type A personality and the Big Five has been found for extraversion (e.g., *r* = 0.17; Hicks and Mehta, 2018) we also expect at least a positive correlation between laughter frequency and extraversion (maybe also with other personality facets).

Mannell and McMahon (1982) found that more laughter during a day was associated with increased positive mood and decreased negative mood. Vlahovic et al. (2012) found associations with happiness. Cheerfulness (Ruch et al., 1996, 1997) is another concept where an association has been found with laughter in past research (e.g., Duchenne smiles, Auerbach, 2017). Cheerfulness is considered as the basis of good humor and in turn good humor is the basis for laughter (Ruch et al., 2019). Therefore, we expect that higher laughter frequency is associated with higher happiness, higher satisfaction with life, and higher cheerfulness.

But laughing not only has positive aspects. Gelotophobia, for example, is the fear of being laughed at. Research found that people with higher values of gelotophobia have not only a higher fear of being laughed at, but also they experience laughter differently, sometimes even incorrectly. These people judge laughter as being rather unpleasant (e.g., Ruch and Proyer, 2008; Ruch et al., 2014). To our knowledge, it is still unclear if gelotophobia is associated with laughter frequency of people suffering from this fear. If these people see laughter as unpleasant, they might also laugh less frequently during their everyday life.

Research Question 2: Are personality (Big Five), well-being (i.e., happiness, satisfaction with life), cheerfulness, and gelotophobia associated with laughter frequency?

## 2 Methods

### 2.1 Participants and ethics

Participants were recruited through different channels (e.g., word of mouth, students), constituting a convenience sample. To determine the number of participants needed to reach a power of 80%, we performed a power analysis. The study that is closest to our design is Martin and Kuiper (1999). They present small to medium effect sizes for their results (gender-differences in laughter frequency: *d* = 0.46; correlations of laughter frequency with age, humor scales, and Type A personality |*r*| = 0.13–0.38). This would result in sample sizes from 63 to 364 participants to reach a power of 80% (one-tailed, *α* = 0.05, sample ratio 1:1, *ρ* = 0.0).

In a first step, a methodological study analyzed whether the usage of a wearable is indeed better than a smartphone (experimental design, for details see Volsa et al., 2023). In sum, 140 participants started the study (75 smartphone groups and 65 wearable groups). Because Volsa et al. (2023) could indeed show that due to the higher participant burden in the smartphone group compared to the wearable group, smartphone users often did not log a laughter event, data from smartphone users were excluded from further analyses. Out of the 65 participants, six participants had to be excluded due to technical problems and seven due to a misunderstanding of the exact assessment procedure (e.g., using a −90° to +90° scale with the PAS or switching the anchor points of the PAS). The remaining participants produced 9,293 single assessments of which 32 (0.4%) had to be deleted due to a button count number > 3 (final *k* = 9,261 single assessment incidents), which should not be possible due to the assessment instruction (belly laugh = single button press; fit of laughter = double button press; and time-based baseline measurements = triple button press).

Participants were on average 29.2 years old (*SD* = 12.61, range 18–74 years) and predominantly women (67.3%; the remainder identified as men). The wearable was predominantly used on the left hand (69.0%) without changing the position during the 4-week assessment phase (98.1%).

The study was conducted in accordance with the Declaration of Helsinki, the standards of the German and Austrian Psychological Societies the American Psychological Association, as well as the guidelines of the Department of Psychology and Psychodynamics, Karl Landsteiner University of Health Sciences. The study was non-invasive, did not include institutionalized participants (e.g., patients), participation was voluntary, and all participants were 18 years of age or older. Participants were mostly university/college students recruited by us. Participation was completely voluntary and anonymous; participants had the right to withdraw at any time during the study without penalty. Therefore, the study was exempt from a formal approval based on the author’s research institution (i.e., waiver policy). As an incentive, participants had the option to enter a raffle after the completion of the study. In this raffle, we randomly chose 30 participants to each receive €200.

### 2.2 Measures

#### 2.2.1 Wearable: daily questions

Participants were instructed to log their current happiness using the wearable either when they were prompted by a haptic signal (i.e., vibration of the wearable using time-based sampling), or whenever they experienced a laughter event (i.e., event-based sampling). Happiness was measured using a PAS (see Section 2.3) ranging from “not happy” (horizontal position) to “very happy” (vertical position). Furthermore, laughter events were categorized either as a “belly laugh” or a “fit of laughter.” Participants indicated the type of measurement with the number of consecutive button-presses, i.e., one press for belly laughs, two for a fit of laughter, and three for reacting to a signal (i.e., randomized time-based assessments of happiness as baseline measure). The PAS was already validated in a previous study (Stieger et al., 2020). There, based on two pilot studies (4-week field study and lab study) and data from a 2-week ESM study on social media ostracism (i.e., *N* = 53 participants and 2,272 event- and time-based assessments), PAS angles were found to be accurate and reliable (eight-item extraversion measure: Cronbach *α* = 0.83), and the Visual Analogue Scale (VAS) and PAS values were highly correlated, suggesting that the PAS is a valid measurement procedure for assessing fleeting and/or frequent micro-situations in everyday life.

#### 2.2.2 Online: final survey

After completing the longitudinal phase of the study (see Section 2.4), participants filled out a questionnaire which included items about their demographics as well as the following concepts. The final questionnaire was presented online via the SoSci Survey platform.^1^

##### 2.2.2.1 Personality

We assessed the classical Big Five personality traits by using the BFI (44 items; John et al., 1991; German version by Lang et al., 2001). All subscales were assessed on five-point Likert scale, ranging from 1 (strongly disagree) to 5 (strongly agree). Internal consistency for each subscale was as follows: extraversion (eight items, Cronbach *α* = 0.87), agreeableness (nine items, *α* = 0.87), conscientiousness (nine items, *α* = 0.86), neuroticism (eight items, *α* = 0.87), and openness (10 items, *α* = 0.78).

##### 2.2.2.2 Satisfaction with life

Life satisfaction was assessed using the Satisfaction with Life Scale (SWLS; Diener et al., 1985; German version by Glaesmer et al., 2011). The scale has five items using a seven-point Likert scale, ranging from 1 (strongly disagree) to 7 (strongly agree). Reliability was *α* = 0.88.

##### 2.2.2.3 Gelotophobia

We assessed gelotophobia with the Gelotophobia Scale (German version: Ruch and Proyer, 2008). Gelotophobia was measured with 15 items using a four-point Likert scale, ranging from 1 (strongly disagree) to 4 (strongly agree). Reliability was α = 0.92.

##### 2.2.2.4 Cheerfulness

The State–Trait Cheerfulness Inventory (STCI) is a self-report instrument measuring three concepts: cheerfulness, seriousness, and bad mood (as a state, as well as a trait; German version: Ruch et al., 1996, 1997). We only used the trait version (STCI-T) to investigate how the three concepts are associated with spontaneous laughter as well as fits of laughter. Each subscale consists of 20 items and uses a four-point Likert scale, ranging from 1 (strongly disagree) to 4 (strongly agree). Reliability for each subscale was as follows: Cheerfulness (*α* = 0.93), seriousness (*α* = 0.75), and bad mood (*α* = 0.96).

### 2.3 Wearable

We used a commercially available, wrist-worn, one-button wearable for data collection. An open-source Android application allows programming these devices for ESM designs (https://github.com/KL-Psychological-Methodology/ESM-Board-Admin; Volsa et al., 2023). Data are stored offline on the device and are retrieved at the end of the study via a Bluetooth connection using the same application.

To assess happiness in a more fine-grained manner, we used a PAS (Stieger et al., 2020; Volsa et al., 2023). Similar to a Visual Analogue Scale, the PAS uses two set end points and divides the space between them in a gradual fashion. Using data from the wearable’s accelerometer, the orientation of the participant’s lower arm during the button press is inferred; thereby indicating their happiness between “not happy” (0°, i.e., horizontal lower arm position) and “very happy” (90°, i.e., lower arm in upright position). To maintain comparability with a previous study that used these data for validating the PAS (Volsa et al., 2023), the resulting degree range from 0 to 90° was then rescaled to a value range from 0 to 100.

### 2.4 Design and procedure

The design of the present laughter study was largely based on event-contingent ESM studies from the past, supplemented by time-contingent sampling to gain insights about situations where participants are not laughing. We used a modified version of the daily laughter record (DLR) initially introduced by Martin and Kuiper (1999) by applying the following specifications: (1) We only assessed the strength of mirth by differentiating between normal spontaneous laughter (i.e., belly laugh; a single button click on the wearable) and a laughter fit (two clicks). We did not ask for the source of laughter (mass media, spontaneous situation, joke, and event) and whether others were present when laughing occurred because of technical restrictions of the wearable (wearable only has a single button); (2) However, we asked participants to rate their happiness by using the accelerometer angle measurement procedure described above (click of the wearable’s button after the arm was put into the desired position/angle; PAS). This is an amendment to the DLR, which should yield more information about the impact of laughing on well-being or the reverse effect, if applicable (for a review, see Devereux and Heffner, 2007; Hofmann and Ruch, 2017). (3) Furthermore, to set a standard of reference to evaluate the laughter incidents against, participants were additionally prompted by the coin vibration motor to rate their baseline happiness at random times (time-based sampling). The wearable was programmed to elicit signals in three predefined time frames per day (for a similar procedure on smoking, see Rathbun et al., 2006). (4) The study lasted for 4 weeks to have a long enough time frame for laughter (especially laughter fits) to occur. (5) At the end of the study, participants were asked to complete a final online questionnaire to assess further relevant psychological constructs, which are of interest when it comes to laughter.

The study took place between July 2019 and January 2022. Participants were introduced to the study in face-to-face meetings. After being provided with an overview of the study procedure, participants signed the informed consent. Participants were then provided with the wearables and information on how to use the devices. They were also given the opportunity to try out the device to familiarize themselves with it.

The 4-week field phase started on the day after the initial meeting. Participants received three pseudo-random notifications each day (in the time frames 9 a.m.–12 p.m., 12 p.m.–3 p.m., and 3 p.m.–6 p.m.), prompting them for a baseline-happiness measurement. They were also instructed to log laughter events in their everyday life whenever these occurred. Following completion of the study after 28 days, participants filled out the final cross-sectional online questionnaire, and returned the wearable in a face-to-face meeting. Participants were then offered the possibility to get personal feedback about their results.

### 2.5 Statistical analyses and data availability

Our data, survey materials, and codes are available on the Open Science Framework at https://osf.io/qg3w5/. Following an initial inspection of the wearable data, we found out that in 779 cases (8.4%), angle measurements used in the PAS, were lower than 0 degrees although participants were instructed only to use angles between 0 and+90 degrees. A graphical inspection of the distribution of the data did not reveal any inconsistencies, i.e., negative values were most probably due to participants not really leveling their measurements to a horizontal level (e.g., when lying in bed or being distracted during assessments; see Supplementary Figure S1). Nevertheless, we also used a winsorized dependent happiness measure (i.e., all negative values were set to 0). This did not change any main outcome of the research questions (see Supplementary Table S1).

We used *R* (R Core Team, 2014) to conduct multi-level models using the *lme4* (Bates et al., 2015) and *sjstats* packages (Lüdecke, 2018). Furthermore, the *effectsize* package for the calculation of standardized effect sizes (Ben-Shachar et al., 2020). Random-intercept, fixed-slope multi-level regression analyses were calculated to analyze the effects of type of laughter (baseline vs. belly laugh vs. fit of laughter; level 1), participant gender, age, personality, gelotophobia, life satisfaction, cheerfulness, seriousness, and bad mood (all level 2) on happiness. Multi-level models accounted for the nested design of our study with measurement occasions (level 1) nested within persons (level 2). All level 2 predictors were grand-mean centered (cgm = centered grand mean) except for participant gender (Enders and Tofighi, 2007; Curran and Bauer, 2011; Nezlek, 2012).

Before adding any predictors, we ran a baseline model to calculate intraclass correlation coefficient (ICC) values (see Table 1). In general, we also considered analyzing possible cross-level interactions between level 2 and level 1 variables. Because the ratio between sample size and number of predictors in the model was rather unfavorable, we refrained from interpreting these interactions. Therefore, in order to avoid the dangers of overfitting and for the sake of a parsimonious model, we did not include cross-level interactions in the final model, which also has the benefit of improving the power of the design. The final model is displayed below:


$$\begin{array}{l} \text{Level}\;1\;\left(\text{within}\;\text{person}\right):{\text{Happiness}}_{ti}=\pi_{0i}+\pi_{1i}\times \text{Belly}\;{\text{laugh}}_{ti}\\ \;+\pi_{2i}\times \text{Fit}\;\text{of}\;{\text{Laughter}}_{ti}+e_{ti} \end{array}$$

$$\begin{array}{l} \text{Level}\;2\;\left(\text{between persons}\right):\pi_{0i}=\\ \beta_{00}+\beta_{01}\times \text{Gender}\left(\text{women}\right)+\beta_{02}\times \text{Age}.cgm+\\ \beta_{03}\times \text{Extraversion}.cgm+\beta_{04}\times \text{Agreeableness}.cgm+\\ \beta_{05}\times \text{Conscientiousness}.cgm+\beta_{06}\times \text{Neuroticism}.cgm+\\ \beta_{07}\times \text{Openness}.cgm+\beta_{08}\times \text{Gelotophobia}.cgm+\\ \beta_{09}\times \text{Life}\;\text{satisfaction}.cgm+\beta_{010}\times \text{STCI}\;\text{bad}\;\text{mood}.cgm+\\ \beta_{011}\times \text{STCI}\;\text{cheerfulness}.cgm+\beta_{012}\times \text{STCI}\;\text{seriousness}.cgm+r_{0i} \end{array}$$

**Table 1:** Results of the multi-level analyses with happiness as the criterion.

	Fixed					Random	
	Coeff.	*B*	*SE*	β	*t*	Coeff.	*SD*
Intercept (Baseline)	β_00_	41.5	6.14		6.75^***^	*r*_0*i*_	19.90
Within-person							
Belly laugh	β_10_	8.6	0.66	0.09	13.01^***^		
Fit of laughter	β_20_	12.9	1.08	0.07	11.91^***^		
Between-person							
Gender (women)	β_01_	−11.5	8.10	−0.28	−1.42		
Age.cgm	β_02_	0.2	0.25	0.14	0.84		
Extraversion.cgm	β_03_	4.4	5.78	0.17	0.76		
Agreeableness.cgm	β_04_	1.9	7.31	0.05	0.26		
Conscientiousness.cgm	β_05_	2.1	6.53	0.07	0.32		
Neuroticism.cgm	β_06_	10.3	6.85	0.40	1.50		
Openness.cgm	β_07_	1.8	6.00	0.06	0.31		
Gelotophobia.cgm	β_08_	−0.1	6.89	<0.01	−0.02		
Life Satisfaction.cgm	β_09_	1.1	3.62	0.06	0.31		
STCI—bad mood.cgm	β_010_	−3.6	9.48	−0.12	−0.39		
STCI—cheerfulness.cgm	β_011_	4.8	9.82	0.13	0.49		
STCI—seriousness.cgm	β_012_	−8.4	12.53	−0.14	−0.67		
*R*^2^_conditional_ = 43%, *R*^2^_marginal_ = 8%; ICC = 36%							

Reference category for belly laugh and fit of laughter were the (time-based) baseline assessments. Reference for gender was men. ^*^p < 0.05, ^**^p < 0.01, and ^***^p < 0.001. ICC of the null model. cgm, Centered around the grand mean; β, Standardized B.

We used *R2GLMM* (Nakagawa and Schielzeth, 2013; Nakagawa et al., 2017) as a measure of explained variance, which can be interpreted like the traditional *R*^2^ statistic in regression analyses. *R*^2^_conditional_ represents the proportion of variance explained by both fixed and random factors and *R*^2^_marginal_ the proportion of variance explained by the fixed factors alone.

Furthermore, we used the *reReg* package in *R* (Chiou et al., 2023) to perform regression analysis of recurrent events (i.e., predictors of laughter frequency, see Research question 2). The package implements a variety of models that can accommodate different model forms for the recurrent event process. We chose the Cox-type proportional rate model (Lin et al., 2000), as the model form for the recurrent event process without an informative terminal event. The model assumes that the intensity function of recurrent events depends on a set of covariates and uses weighted pseudo-partial score equations (e.g., the cumulative baseline rate) to account for dependency among recurrent events within a subject (Huang and Huang, 2022). This estimation approach abandons the strong assumption of frailty models that dependence between recurrent events can be described by one explicit latent parameter and it has been shown to provide a more efficient estimation of model parameters (Huang and Huang, 2022). For each subject, the rate function of laughter is defined as


$$\lambda \left(t\right)=\lambda_{0}\left(t\right)\exp\left(X^{⊤}\beta \right)$$

where λ_0_ is the baseline rate function, *X* represents the transposed matrix (i.e., ^⊤^) of covariates, β is a vector of parameters, and *t* is the time in the study. For this analysis, events of belly laughter and fit of laughter were pooled together.

## 3 Results

### 3.1 Descriptives

**Figure 1:**
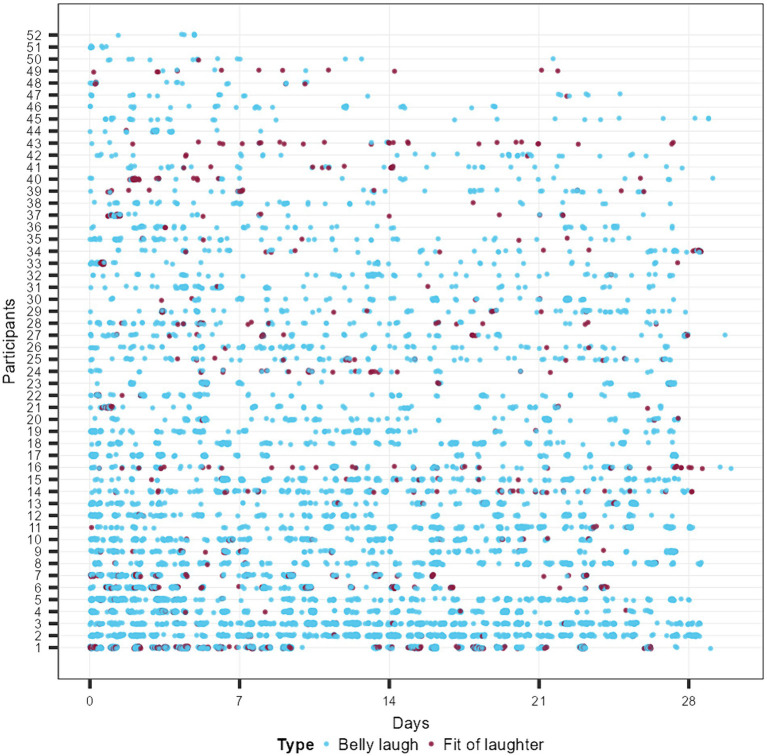
Recorded laughing events (*n* = 6,767) across all study participants (*N* = 52) during participation. Belly laughs are shown in blue, while fits of laughter are shown in red.

In sum, each participant recorded 155 belly laughs on average during the 4-week time frame (Median = 69; *SD* = 125.98, range 6–556). This represents an average number of 2.5–5.5 belly laughs per day (depending, if Mean or Median is used). As expected, fits of laughter were rare compared to belly laughs with 16 fits of laughter on average during the assessment phase (Median = 7.5; *SD* = 33.30, range 0–228). This represents about a single fit of laughter every second to fourth day on average (0.57 and 0.27 per day, depending, if Median or Mean is used). Furthermore, on average participants answered 46 time-based baseline measurements during the 4 weeks (*SD* = 27.7) out of the 56 time-based baseline assessments elicited by the ESM design during the 4 weeks. For an overview of all laughter incidences (*k*_laughter_ = 6,767) over time, see Figure 1.

As can be seen in Supplementary Figure S2, there was a trend for laughter frequency over the time of the day, i.e., laughter frequency clearly increased during the day replicating past research (Martin and Kuiper, 1999). This applied to belly laughs and fit of laughter similarly. Furthermore, as can be seen in Supplementary Figure S2, the baseline assessments were almost equally distributed throughout the day, speaking for the successful randomness of eliciting time-based notifications through the wearable itself (between 9 a.m. and 6 p.m.). Furthermore, the present results also replicate past research on laughter throughout the week (Helliwell and Wang, 2014). Laughter frequency was lower on Monday–Thursday, but higher from Friday to Sunday (Supplementary Figure S3). On Fridays, belly laughs and fit of laughter frequencies were highest (probably anticipating the forthcoming weekend). Again, time-based assessments were quite uniform across days with slightly higher counts on weekdays compared to weekends (maybe due to participants missing some notifications due to longer sleeping hours, or in general less commitment for the study’s procedure).

### 3.2 RQ1: is happiness higher when laughing compared to when not laughing?

Happiness ratings were on average 38.5 during baseline assessments. As expected, a belly laugh significantly raised happiness (by 8.6 points on the PAS) as well as did a fit of laughter (by 12.9 points on the PAS; see Table 1). Although these differences were significant, none of the level 2 variables reached statistical significance. Overall, the results speak for the validity of the data and used procedure (wearables using the PAS; see also Volsa et al., 2023).

### 3.3 RQ2: are personality, well-being (happiness, satisfaction with life), cheerfulness, and gelotophobia associated with laughter frequency?

We employed recurrent event regression to investigate the relationship between the *frequency* of laughing events and individual differences in personality traits: cheerfulness, happiness, gelotophobia, life satisfaction, time of day, age, and gender. We first tested the *Time of Day* variable as a continuous predictor (i.e., actual time the laughter event took place in hours), but found two issues: (1) it was not linearly-related to the hazard rate of laughter events, and (2) it violated the proportional rate assumption, meaning that the rate for different hours of the day varied over time potentially biasing the estimated parameters (Supplementary Figure S4). Transforming the *Time of Day* variable into a cyclic variable using cosine and sine functions, we found that the cyclic term was indeed significant (Supplementary Table S2). However, while this could account for non-linearity, this did not solve the problem of non-proportionality. We then tested an alternative model with *Time of Day* as a categorical predictor, dividing the 24 h into three 8-h bins based on the distribution of laughter events over the day. The bins were aligned with the time window of the baseline measurements starting at 9 a.m. Visual inspection of the event distribution across the day corroborated the choice of three phases, showing a night phase from 1 a.m. to 9 a.m., a working phase from 9 a.m. to 5 p.m., and an evening phase from 5 p.m. to 1 a.m (Supplementary Figure S5).

**Figure 2:**
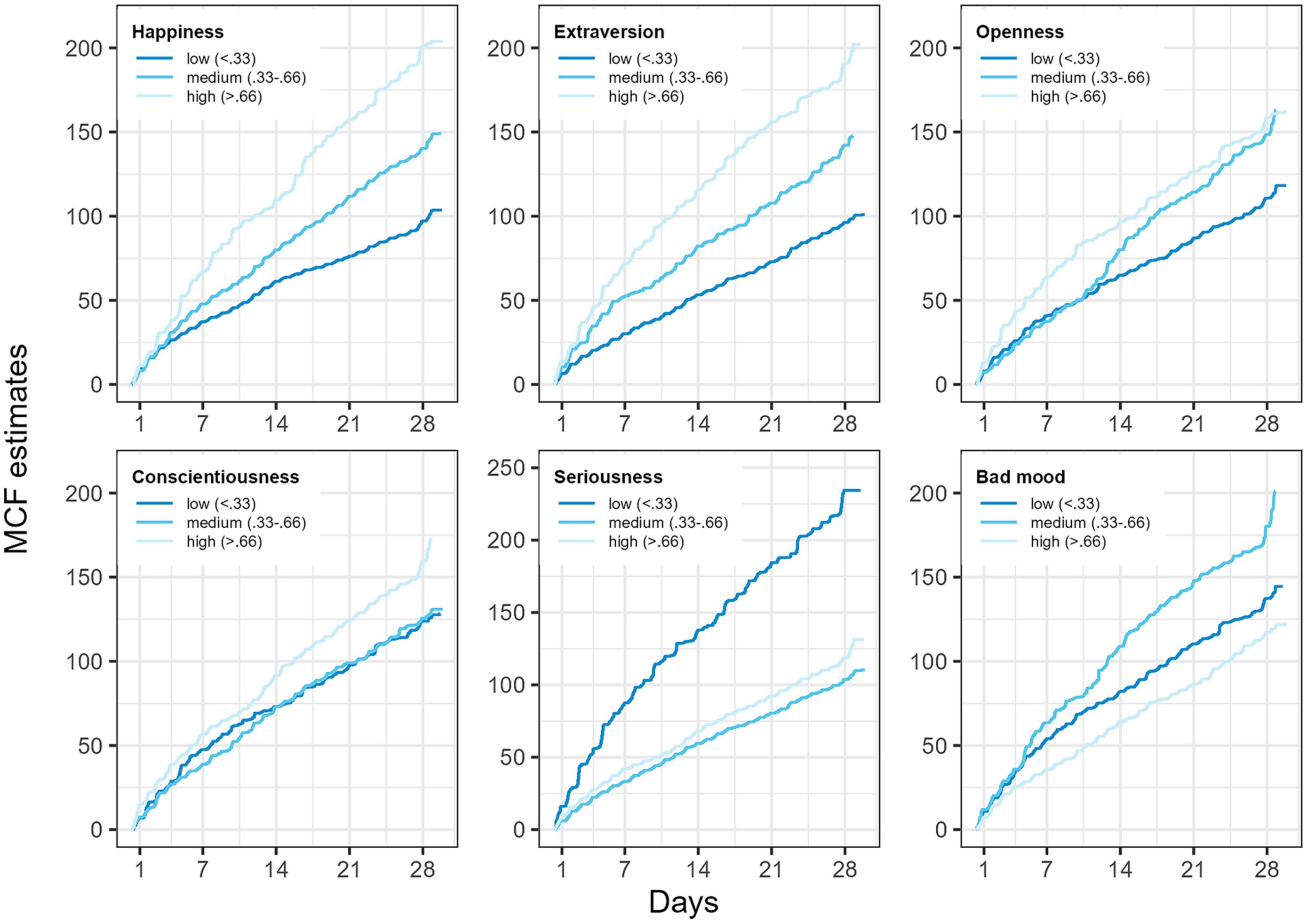
Non-parametric Nelson-Aalen estimates (Lawless and Nadeau, 1995) of the mean cumulative function (MCF) of laughing events for different levels (defined at 33 and 66% percentiles for three levels) of happiness, personality traits, seriousness, and bad mood across the study period. Higher scores on happiness, extraversion, openness, and conscientiousness, and in contrast lower scores on seriousness, correspond to increased rates of laughter across days.

Our results revealed a significant positive association between the frequency of laughing events and happiness (*p* < 0.001), and the personality traits openness (*p* < 0.001), and conscientiousness (*p* = 0.009, Table 2). Specifically, individuals who reported more frequent laughing events also reported higher levels of happiness compared to those who reported fewer laughing events replicating the results from above (i.e., Research question 1). In addition, we found that seriousness was significantly negatively related to laughing frequency (*p* = 0.008), whereas bad mood (*p* = 0.017) and life satisfaction (*p* = 0.014) had a positive effect on laughing frequency (for an overview, see also Figure 2).

**Table 2:** Results of the recurrent event regression analysis with laughter frequency (belly and fit of laughter) as the criterion.

	Fixed			
	Coeff.	*B*	*SE*	*z*
Gender	β_1_	0.76	0.23	3.35^***^
Age.cgm	β_2_	−1.75	0.24	−7.16^***^
Time of day (9 a.m.–5 p.m.)	β_3_	0.35	0.27	1.31
Time of day (5 p.m.–1 a.m.)	β_4_	−0.13	0.30	−0.42
Happiness.cgm	β_5_	0.25	0.02	13.21^***^
Openness.cgm	β_6_	0.23	0.05	4.46^***^
Agreeableness.cgm	β_7_	−0.03	0.06	−0.55
Conscientiousness.cgm	β_8_	0.15	0.06	2.59^**^
Extraversion.cgm	β_9_	0.08	0.08	1.03
Neuroticism.cgm	β_10_	0.03	0.12	0.26
Gelotophobia.cgm	β_11_	−0.16	0.08	−1.91
Life Satisfaction.cgm	β_12_	0.18	0.07	2.46^*^
STCI—bad mood.cgm	β_13_	0.29	0.12	2.37^*^
STCI—cheerfulness.cgm	β_14_	0.17	0.10	1.78
STCI—seriousness.cgm	β_15_	−0.22	0.08	−2.62^**^
Gender:Age.cgm	β_16_	1.07	0.13	8.18^***^
Gender:Time of day (9 a.m.–5 p.m.)	β_17_	−0.18	0.15	−1.18
Gender:Time of day (5 p.m.–1 a.m.)	β_18_	0.15	0.17	0.88

*N* = 52, *n*_recurrent events_ = 6,767, mean recurrent laughing events per subject = 130.1. Reference for gender was men. Reference for Time of Day were the night hours 1 a.m.–9 a.m.; ^***^p < 0.001, ^**^p < 0.01, and ^*^p < 0.05. cgm, Centered around the grand mean. As *B* represents log odds ratios, effects can be interpreted as small (0.36), medium (0.90), and large (1.45; Sánchez-Meca et al., 2003).

**Figure 3:**
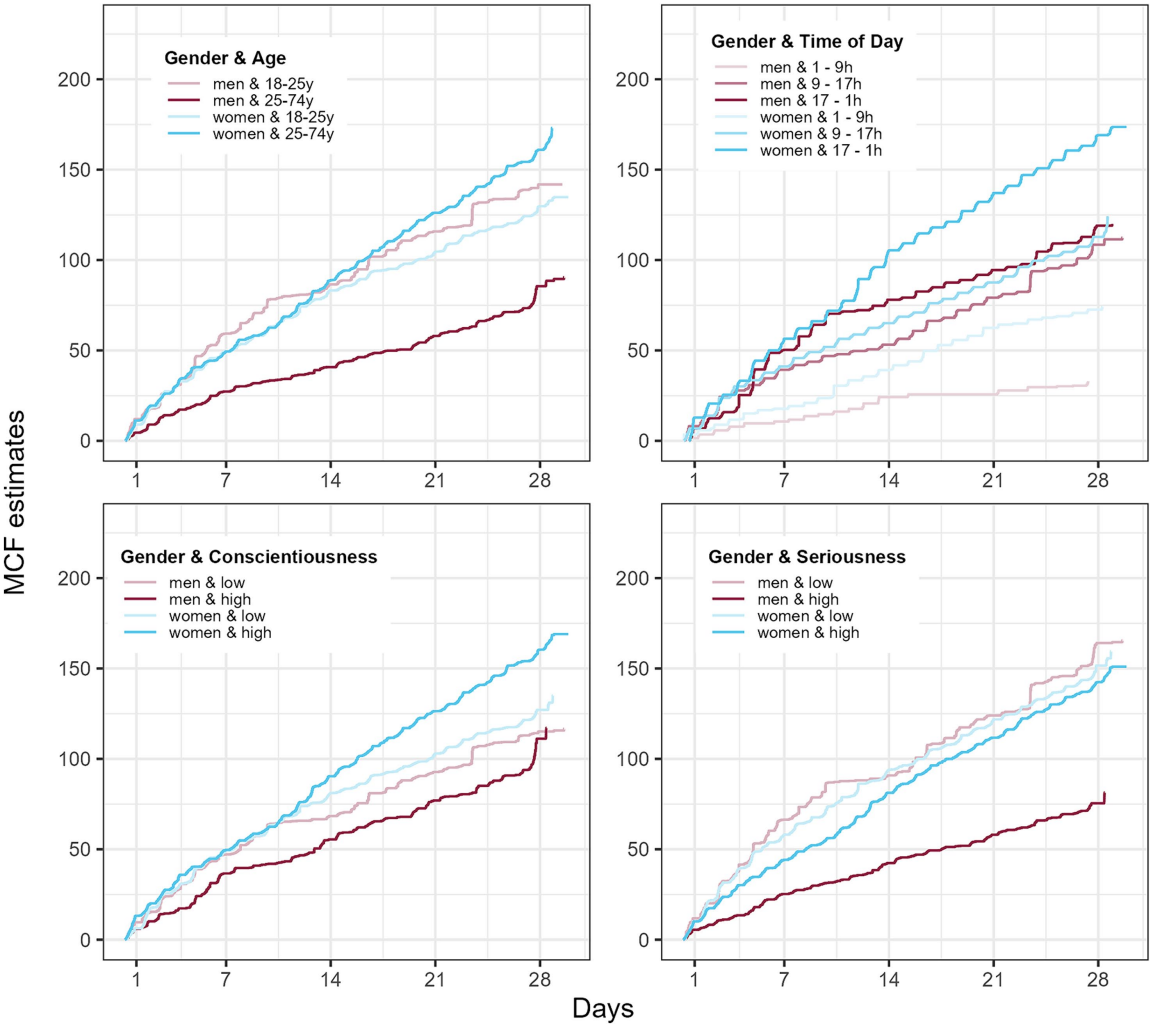
The mean cumulative function (MCF) of laughing events for different levels (defined at median for two levels or 33 and 66% percentiles for three levels, respectively). Women tend to have lower frequency when they are older, whereas men show an increase in laughing frequency with age. Moreover, women showed a significant increase in laughter with time of day. Additional analyses separated by gender also hinted on possible gender-by-conscientiousness and gender-by-seriousness interaction effects on laughing frequency (Supplementary Tables S2, S3).

In addition, we found that women exhibited a higher laughing frequency (main effect of gender, *p* < 0.001), and a gender-by-age interaction (*p* < 0.001, Table 2; Supplementary Figures S2, S3). Closer assessment of the interaction effect revealed that women have a higher frequency of laughing when they are older, while men show a decrease of laughing frequency with age (Figure 3). We found no significant gender-by-time of day interactions (*p* > 0.23). This largely corroborates findings of Martin and Kuiper (1999) who reported a gender effect descriptively in the same direction (women > men).

Although past research found a positive association between cheerfulness and the frequency of Duchenne smiles (Auerbach, 2017), we could not replicate this result when it comes to belly laughs and fits of laughter in the full sample. However, a separate analysis for men and women showed that cheerfulness was only a significant predictor of laughing frequency of men (Supplementary Tables S2, S3). Furthermore, we could not find any significant association between laughter frequency and gelotophobia (see Table 2); although for women, it was descriptively in the assumed direction (i.e., negative association) and almost significant (Supplementary Table S4). The gender-specific result must be interpreted with caution due to the uneven balance of men and women in the sample, which could affect power.

Results of additional analyses for separate laughing types of belly laughter and fit of laughter are shown in Supplementary Tables S5, S6.

## 4 Discussion

In the present study, we examined laughter in everyday life by using new measurement techniques such as one-button wearables worn on the wrist and a body-posture-based measurement scale using the accelerometer. These techniques should allow an easy measurement in the field without disturbing participants much in their everyday lives (Volsa et al., 2022, 2023). This should result in good data quality without biases from memory (because laughter events are assessed in the moment when they happened), motivation (postponing or missing the assessment), and mode of implementation (wearables only slightly disturb participants during their everyday lives; measurement with the PAS is very intuitive and easy to do; Volsa et al., 2023).

In line with past research, laughter was a frequent event in participant’s everyday life. Participants had a belly laugh on average 2.5 times per day, and approximately once every 4 days, a belly laugh led to a fit of laughter. As expected, during a belly laugh, participants’ happiness was 8.6 points higher on the PAS compared to the baseline measurements, and a fit of laughter even raised happiness by 12.9 points on the PAS (PAS range 0–100; Vlahovic et al., 2012). Both results also speak for the validity of the measurement procedure (wearable and PAS). Laughter frequency is very much in line with the results of Kuiper and Martin (1998) and Martin and Kuiper (1999) who reported on average 18 laughs per day from participants, but in their study also other forms of laughter were recorded (e.g., smiling) by participants and the assessment phase was only 3 days. Furthermore, Valeri (2006) found on average 19 laughs per day using the DLR like Martin and Kuiper (1999).

Similarly, in line with Martin and Kuiper (1999), laughter frequency increased from morning to evening (Supplementary Figure S2). Because social contacts usually also increase over the day (especially fun and active social interaction: Vittengl and Holt, 1998) and laughter often happens in social situations, the laughter increase probably reflects the increase in social situations. Similarly, we did find gender-differences in laughter frequency across men and women (although descriptively in the same direction but not significant in Martin and Kuiper, 1999). Women laughed slightly more than men (women: Median = 2.64 laughs per day; men: Median = 2.25 laughs per day). Furthermore, laughter frequency was negatively correlated with participants’ age, i.e., the older, the higher the laughter frequency (again, in line with Martin and Kuiper, 1999).

Regarding personality, participants who were more conscientious and open had a higher laughter frequency compared to those who were more unconscientious and reserved. Because open people also have a higher social contact frequency and because laughter usually happens in social interactions, this association seems comprehensible and in line with Martin and Kuiper (1999), who found an association with the Type A personality, which is also correlated with openness (Hicks and Mehta, 2018).

Interestingly, although laughter frequency was positively associated with happiness (Vlahovic et al., 2012) and life satisfaction, we did not find a significant association with cheerfulness, though this association was descriptively positive, as assumed cheerfulness is considered to be the basis of good humor, and, in turn, good humor should be the basis for laughter (Auerbach, 2017; Ruch et al., 2019). Regarding bad mood, unexpectedly, being in a generally bad mood increased the laughing frequency on average in our participants. However, this effect seems to be driven by participants scoring medium in bad mood, whereas participants scoring highest and lowest in bad mood also laughed the least (Figure 2), i.e., there seems to be a non-linear association between laughter frequency and bad mood as a trait. Furthermore, it is crucial to consider that situational characteristics and dynamics of when a laughter event occurs plays a role. For example, instances of laughter may not always reflect cheerfulness, particularly in social situations where laughter is a form of social norm (e.g., laughing about jokes made by one’s superior out of social etiquette or using laughing to identify norm violations and to sanction others’ behavior, either directly or through sarcasm). Therefore, associations between laughter frequency and cheerfulness could be weakened. Nevertheless, due to the modest statistical power of our study, we can only speculate about the reasons for this. However, exploring these factors could be also a fruitful direction for future research.

Furthermore, we found that participants with signs of gelotophobia laughed less often than non-gelotophobe participants. It seems that participants who are fearful of being laughed at by others in return also less-often laugh, although the effect failed to reach statistical significance. Gelotophobic people not only have a higher fear of being laughed at, they also experience laughter differently, sometimes even incorrectly (e.g., Ruch and Proyer, 2008; Ruch et al., 2014). If these people really show a different pattern of laughing (e.g., more laughing when alone; less happy when laughing), this might be a good approach for future research and could add to the understanding of gelotophobia.

Another interesting aspect for future research would be testing both, behavioral inclinations to laughter as well as gelotophobia on a daily basis. Brauer and Proyer (2020) introduced a behavioral record intended for usage in situational and diary assessments on a daily basis. This might allow to identify whether gelotophobic state-like expressions co-vary with laughter frequency and might provide a more precise estimate of gelotophobia in daily life, including its fluctuations.

### 4.1 Limitations

Several limitations of the present study should be considered. First, due to the measurement procedure of using one-button wearables, we were only able to assess a limited amount of information at each assessment occasion. It would also have been of interest to assess the background of the laughing event, such as if laughing occurred while being alone or with others. Wearables with a screen would be beneficial to assess multiple items (for a recent development, see Volsa et al., 2022). Second, we focused only on two forms of laughing—a belly laugh, and a fit of laughter. The main reason for this was the event-based nature of the study. Participants have to be aware of the kind of laughter in order to do the assessment self-paced. This might be difficult for laughter events, which are rather unconscious (e.g., social smiling). Third, although our sample was community-based, still more-than-expected students were in our sample, i.e., a more diverse sample might come to different conclusions, even though we could successfully replicate several results from Martin and Kuiper (1999) who used a community-based sample (recruitment through newspaper and cable TV advertisements). Fourth, the data collection took place during the COVID pandemic, which might have influenced the outcome of the study, i.e., it is unclear if these results are representative for non-pandemic situations. Finally, based on the found effect sizes in Martin and Kuiper (1999), the results regarding interactions or group differences (age, gender) might be slightly underpowered, i.e., these results must be interpreted with caution.

### 4.2 Conclusion

In the present study, we successfully applied a new assessment method using wrist-worn, one-button wearables and a PAS to analyze laughter in everyday life and its frequency associations. We used an experience sampling method design for 4 weeks to enhance the generalizability of our results. By utilizing state-of-the-art statistical methods (i.e., recurrent event regression) for the event-based, multi-level data structure, we could not only replicate past results (Martin and Kuiper, 1999; Vettin and Todt, 2004; Valeri, 2006; Vlahovic et al., 2012) but also pave the way for future research into laughter. Combining innovative statistical and assessment methodologies together with longitudinal designs has the potential to advance our understanding of the reasons behind laughter.

## Data availability statement

The datasets presented in this study can be found in online repositories. The names of the repository/repositories and accession number(s) can be found at: https://osf.io/qg3w5.

## Ethics statement

Ethical approval was not required for the studies involving humans because the study was conducted in accordance with the Declaration of Helsinki, the standards of the German and Austrian Psychological Societies the American Psychological Association, as well as the guidelines of the Department of Psychology and Psychodynamics, Karl Landsteiner University of Health Sciences. The study was non-invasive, did not include institutionalized participants (e.g., patients), participation was voluntary, and all participants were 18 years of age or older. Participants were mostly university/college students recruited by us. Participation was completely voluntary and anonymous; participants had the right to withdraw at any time during the study without penalty. Therefore, the study was exempt from a formal approval based on the author’s research institution (i.e., waiver policy). The studies were conducted in accordance with the local legislation and institutional requirements. The participants provided their written informed consent to participate in this study.

## Author contributions

SS: Conceptualization, Formal analysis, Funding acquisition, Methodology, Resources, Supervision, Visualization, Writing – original draft, Writing – review & editing. SV: Investigation, Methodology, Project administration, Writing – review & editing. DW: Formal analysis, Visualization, Writing – review & editing. DL: Software, Writing – review & editing. BB: Supervision, Writing – review & editing.

## Footnotes

^1^ soscisurvey.de
