# Supplementary material for: Laughter in everyday life: an event-based experience sampling method study using wrist-worn wearables
Source: Front Psychol. 2024 May 2;15:1296955. doi: 10.3389/fpsyg.2024.1296955 (PMC11096579; doi:10.3389/fpsyg.2024.1296955)
Supplement: Supplementary file 1 [file Table_1.DOCX]

Supplementary Material

# Supplementary Figures and Tables

This online supplement contains the following information:

- **Supplementary Figure S1**: Distribution of the happiness ratings from the Physical Analogue Scale (spreaded -100 to +100).
- **Supplementary Figure S2**: Frequency of laughter events and baseline measurements over the course of day.
- **Supplementary Figure S3**: Frequency of laughter events and baseline measurements over the course of a week including a 95% confidence interval.
- **Supplementary Figure S4**: Proportional rates varied over the study period with the Time of Day (hours) – as seen in widely differing slopes of the mean cumulative function (MCF) – violating the proportional rate assumption and potentially biasing the parameter estimate.
- **Supplementary Figure S5**: Frequency of laughter events over the course of a day for each gender for each hour (left) and aggregated phases (right). Points represent the mean, bars depict the standard deviation. There is a considerable within-subject and between-subject variability over the course of the day.
- **Supplementary Table S1**: Results of the Multi-Level Analyses using winsorized happiness scores.
- **Supplementary Table S2**: Results of the recurrent event regression analysis with laughter frequency (belly and fit of laughter) as the criterion with standardized predictors – cyclical predictors.
- **Supplementary Table S3**: Results of the recurrent event regression analysis with laughter frequency (belly and fit of laughter) as the criterion only for men.
- **Supplementary Table S4**: Results of the recurrent event regression analysis with laughter frequency (belly and fit of laughter) as the criterion only for women.
- **Supplementary Table S5**: Results of the recurrent event regression analysis with frequency of belly laughter as the criterion.
- **Supplementary Table S6**: Results of the recurrent event regression analysis with frequency of fit of laughter as the criterion.

## Supplementary Figures


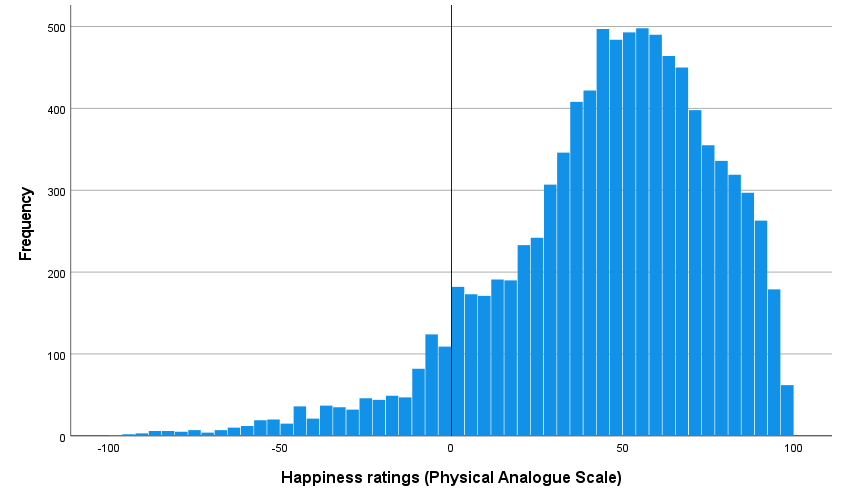


**Supplementary Figure S1.** Distribution of the happiness ratings from the Physical Analogue Scale (spread -100 to +100).


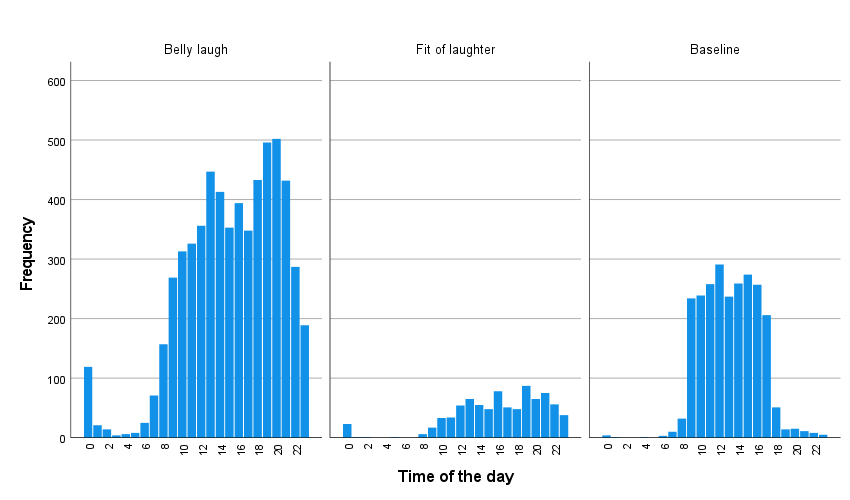


**Supplementary Figure S2.** Frequency of laughter events and baseline measurements over the course of day.


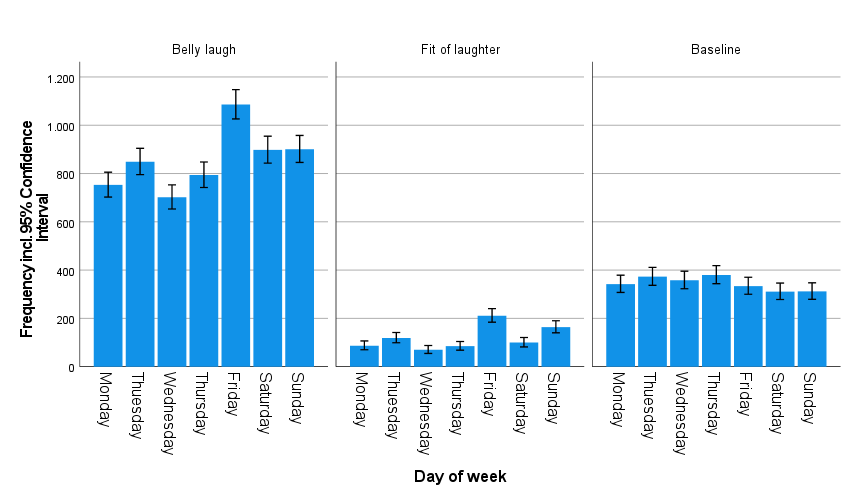


**Supplementary Figure S3.** Frequency of laughter events and baseline measurements over the course of a week including a 95% confidence interval.

**
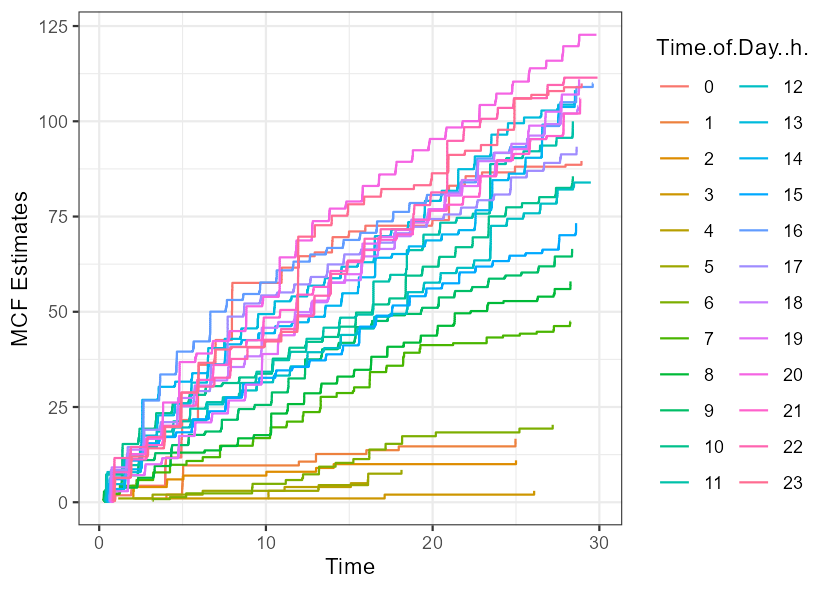
**

**Supplementary Figure S4.** Proportional rates varied over the study period with the Time of Day (hours) – as seen in widely differing slopes of the mean cumulative function (MCF) – violating the proportional rate assumption and potentially biasing the parameter estimate.

**
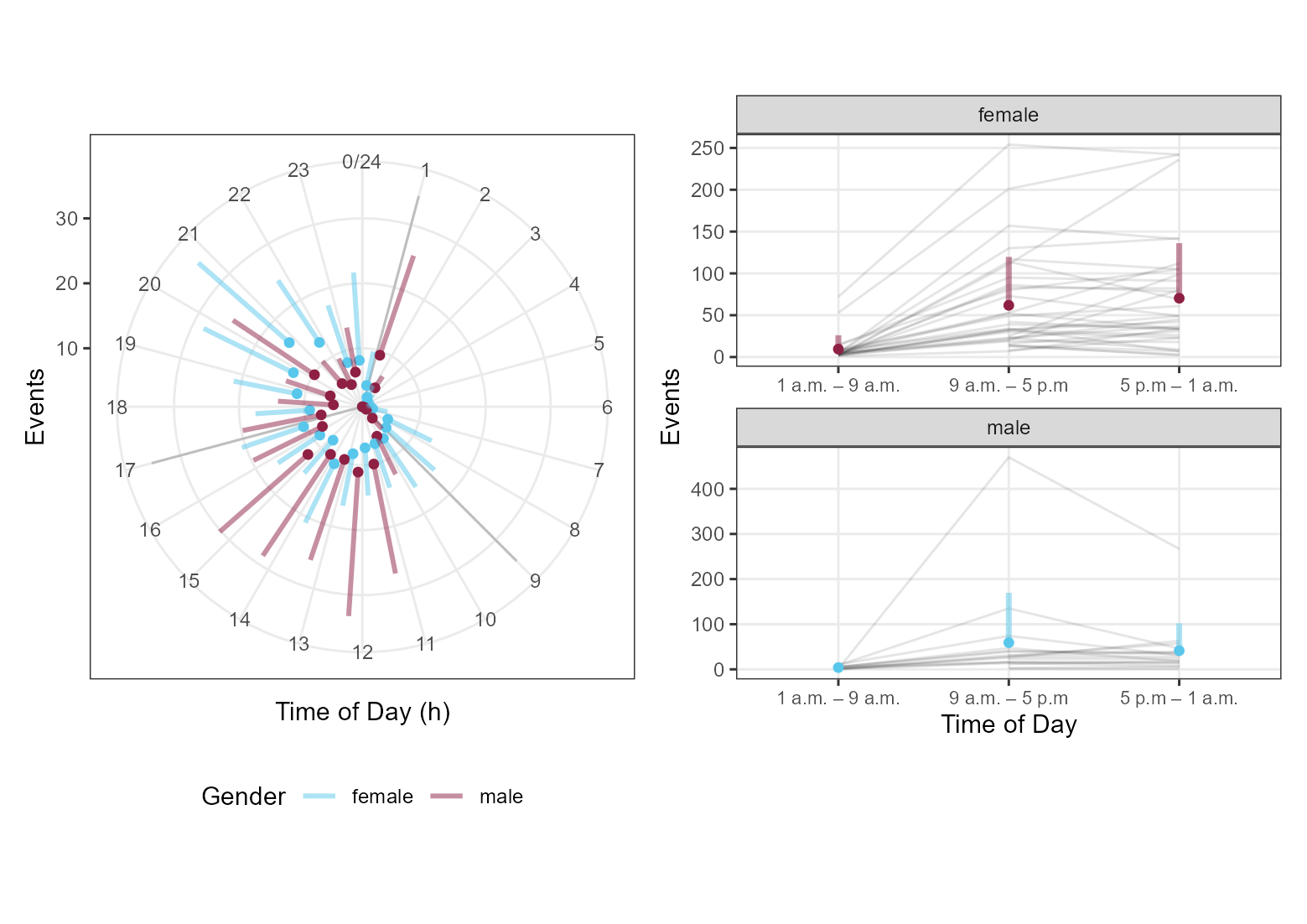
**

**Supplementary Figure S5.** Frequency of laughter events over the course of a day for each gender for each hour (left) and aggregated phases (right). Points represent the mean, bars depict the standard deviation. There is a considerable within-subject and between-subject variability over the course of the day.

## Supplementary Tables

**Supplementary Table S1**. Results of the Multi-Level Analyses using winsorized happiness scores.

|  | Fixed | | | |  | Random | |
| --- | --- | --- | --- | --- | --- | --- | --- |
|  | Coeff. | *B* | *SE* | *t* |  | Coeff. | *SD* |
| Intercept (Baseline) | β_00_ | 40.0 | 4.92 | 8.13*** |  | *r*_0i_ | 14.98 |
| Within-person |  |  |  |  |  |  |  |
| Belly laugh | β_10_ | 8.5 | 0.57 | 15.02*** |  |  |  |
| Fit of laughter | β_20_ | 13.2 | 0.93 | 14.22*** |  |  |  |
| Between-person |  |  |  |  |  |  |  |
| Gender (women) | β_01_ | -5.6 | 6.58 | -0.85 |  |  |  |
| Age.cgm | β_02_ | 0.2 | 0.19 | 1.13 |  |  |  |
| Extraversion.cgm | β_03_ | 4.3 | 4.44 | 0.97 |  |  |  |
| Agreeableness.cgm | β_04_ | 2.5 | 5.56 | 0.46 |  |  |  |
| Conscientiousness.cgm | β_05_ | 1.8 | 4.71 | 0.37 |  |  |  |
| Neuroticism.cgm | β_06_ | 8.5 | 5.05 | 1.68 |  |  |  |
| Openness.cgm | β_07_ | 9.6 | 6.80 | 1.41 |  |  |  |
| Gelotophobia.cgm | β_08_ | -0.3 | 5.22 | -0.05 |  |  |  |
| Life Satisfaction.cgm | β_09_ | 1.3 | 2.72 | 0.49 |  |  |  |
| STCI – bad mood.cgm | β_10_ | -4.1 | 6.76 | -0.61 |  |  |  |
| STCI – cheerfulness.cgm | β_11_ | -0.5 | 7.73 | -0.06 |  |  |  |
| STCI – seriousness.cgm | β_12_ | -10.3 | 9.17 | -1.12 |  |  |  |
| *R*^2^*_conditional_* = 40%, *R*^2^*_marginal_* = 12%; ICC = 33% | | | | | | | |

Note. Reference category for belly laugh and fit of laughter were the (time-based) baseline assessments. Reference for gender was men. ICC of the null model. cgm = centered around the grand mean. * *p* < .05, ** *p* < .01, *** *p* < .001.

**Supplementary Table S2.** Results of the recurrent event regression analysis with laughter frequency (belly and fit of laughter) as the criterion **only for men**.

|  | Fixed | | | |
| --- | --- | --- | --- | --- |
|  | Coeff. | *B* | *SE* | *z* |
| Age.cgm | β_1_ | -0.56 | 0.10 | -5.42*** |
| Time of Day (9 a.m. – 5 p.m.) | β_2_ | -0.36 | 0.37 | -0.96 |
| Time of Day (5 p.m. – 1 a.m.) | β_3_ | -0.45 | 0.36 | -1.24 |
| Happiness.cgm | β_4_ | 0.29 | 0.09 | 3.44*** |
| Openness.cgm | β_5_ | 0.28 | 0.07 | 4.07 |
| Agreeableness.cgm | β_6_ | -0.29 | 0.12 | -2.43* |
| Conscientiousness.cgm | β_7_ | -0.75 | 0.12 | -6.53*** |
| Extraversion.cgm | β_8_ | -0.26 | 0.18 | -1.47 |
| Neuroticism.cgm | β_9_ | -0.02 | 0.12 | -0.16 |
| Gelotophobia.cgm | β_10_ | -0.03 | 0.17 | -0.17 |
| Life Satisfaction.cgm | β_11_ | 0.55 | 0.12 | 4.72*** |
| STCI – bad mood.cgm | β_12_ | 0.59 | 0.23 | 2.57* |
| STCI – cheerfulness.cgm | β_13_ | 1.04 | 0.19 | 5.61*** |
| STCI – seriousness.cgm | β_14_ | 0.13 | 0.15 | 0.83 |

*Note*. *N* = 17, *n* recurrent events = 1,750, mean recurrent laughing events per subject = 102.9. Reference for Time of Day were the night hours 1 a.m. – 9 a.m. cgm = centered around the grand mean. *** *p* < .001, ** *p* < .01, * *p* < .05.

**Supplementary Table S3.** Results of the recurrent event regression analysis with laughter frequency (belly and fit of laughter) as the criterion **only for women**.

|  | Fixed | | | |
| --- | --- | --- | --- | --- |
|  | Coeff. | *B* | *SE* | *z* |
| Age.cgm | β_1_ | 0.24 | 0.11 | 2.06* |
| Time of Day (9 a.m. – 5 p.m.) | β_2_ | 0.00 | 0.11 | 0.03 |
| Time of Day (5 p.m. – 1 a.m.) | β_3_ | 0.20 | 0.12 | 1.69 |
| Happiness.cgm | β_4_ | 0.22 | 0.03 | 7.91*** |
| Openness.cgm | β_5_ | 0.20 | 0.06 | 3.29** |
| Agreeableness.cgm | β_6_ | 0.08 | 0.06 | 1.41 |
| Conscientiousness.cgm | β_7_ | 0.32 | 0.08 | 3.97*** |
| Extraversion.cgm | β_8_ | 0.14 | 0.09 | 1.59 |
| Neuroticism.cgm | β_9_ | -0.05 | 0.15 | -0.32 |
| Gelotophobia.cgm | β_10_ | -0.20 | 0.14 | -1.48 |
| Life Satisfaction.cgm | β_11_ | 0.02 | 0.07 | 0.27 |
| STCI – bad mood.cgm | β_12_ | 0.38 | 0.12 | 3.27** |
| STCI – cheerfulness.cgm | β_13_ | 0.02 | 0.15 | 0.11 |
| STCI – seriousness.cgm | β_14_ | -0.32 | 0.12 | -2.74** |

*Note*. *N* = 35, *n* recurrent events = 5,017, mean recurrent laughing events per subject = 143.3. Reference for Time of Day were the night hours 1 a.m. – 9 a.m. cgm = centered around the grand mean. *** *p* < .001, ** *p* < .01, * *p* < .05.

**Supplementary Table S4.** Results of the recurrent event regression analysis with **frequency of belly laughter** as the criterion.

|  | Fixed | | | |
| --- | --- | --- | --- | --- |
|  | Coeff. | *B* | *SE* | *z* |
| Gender | β_1_ | 0.54 | 0.26 | 2.08* |
| Age.cgm | β_2_ | -1.46 | 0.23 | -6.22*** |
| Time of Day (9 a.m. – 5 p.m.) | β_3_ | 0.10 | 0.28 | 0.35 |
| Time of Day (5 p.m. – 1 a.m.) | β_4_ | -0.32 | 0.32 | -1.01 |
| Happiness.cgm | β_5_ | 0.29 | 0.02 | 14.03*** |
| Openness.cgm | β_6_ | 0.19 | 0.06 | 3.47*** |
| Agreeableness.cgm | β_7_ | 0.01 | 0.06 | 0.13 |
| Conscientiousness.cgm | β_8_ | 0.20 | 0.06 | 3.11** |
| Extraversion.cgm | β_9_ | 0.06 | 0.07 | 0.87 |
| Neuroticism.cgm | β_10_ | 0.00 | 0.13 | -0.03 |
| Gelotophobia.cgm | β_11_ | -0.18 | 0.09 | -2.02* |
| Life Satisfaction.cgm | β_12_ | 0.23 | 0.07 | 3.08** |
| STCI – bad mood.cgm | β_13_ | 0.41 | 0.12 | 3.36*** |
| STCI – cheerfulness.cgm | β_14_ | 0.14 | 0.10 | 1.31 |
| STCI – seriousness.cgm | β_15_ | -0.28 | 0.10 | -2.87** |
| Gender:Age.cgm | β_16_ | 0.91 | 0.12 | 7.38*** |
| Gender:Time of Day (9 a.m. – 5 p.m.) | β_17_ | -0.02 | 0.16 | -0.11 |
| Gender:Time of Day (5 p.m. – 1 a.m.) | β_18_ | 0.26 | 0.18 | 1.40 |

*Note*. *N* = 52, *n* recurrent events = 5,931, mean recurrent laughing events per subject = 114.1. Reference for gender was men. Reference for Time of Day were the night hours 1 a.m. – 9 a.m. cgm = centered around the grand mean. *** *p* < .001, ** *p* < .01, * *p* < .05.

**Supplementary Table S5.** Results of the recurrent event regression analysis **with frequency of fit of laughter** as the criterion*.*

|  | Fixed | | | |
| --- | --- | --- | --- | --- |
|  | Coeff. | *B* | *SE* | *z* |
| Gender | β_1_ | -0.68 | 0.62 | -1.10 |
| Age.cgm | β_2_ | -2.32 | 1.57 | -1.47 |
| Time of Day (9 a.m. – 5 p.m.) | β_3_ | -0.51 | 1.50 | -0.34 |
| Time of Day (5 p.m. – 1 a.m.) | β_4_ | -2.11 | 1.55 | -1.36 |
| Happiness.cgm | β_5_ | 0.36 | 0.08 | 4.60*** |
| Openness.cgm | β_6_ | 0.38 | 0.09 | 4.30*** |
| Agreeableness.cgm | β_7_ | 0.23 | 0.09 | 2.66** |
| Conscientiousness.cgm | β_8_ | -0.21 | 0.11 | -1.83 |
| Extraversion.cgm | β_9_ | 0.83 | 0.23 | 3.52*** |
| Neuroticism.cgm | β_10_ | -0.12 | 0.14 | -0.87 |
| Gelotophobia.cgm | β_11_ | 0.07 | 0.13 | 0.50 |
| Life Satisfaction.cgm | β_12_ | 0.04 | 0.06 | 0.73 |
| STCI – bad mood.cgm | β_13_ | 0.33 | 0.18 | 1.86 |
| STCI – cheerfulness.cgm | β_14_ | -0.18 | 0.12 | -1.52 |
| STCI – seriousness.cgm | β_15_ | -0.29 | 0.14 | -2.12* |
| Gender:Age.cgm | β_16_ | 1.21 | 0.80 | 1.50 |
| Gender:Time of Day (9 a.m. – 5 p.m.) | β_17_ | 0.39 | 0.80 | 0.49 |
| Gender:Time of Day (5 p.m. – 1 a.m.) | β_18_ | 1.09 | 0.81 | 1.35 |

*Note*. *N* = 46, *n* recurrent events = 790, mean recurrent laughing events per subject = 17.2. Reference for gender was men. Reference for Time of Day were the night hours 1 a.m. – 9 a.m. cgm = centered around the grand mean. *** *p* < .001, ** *p* < .01, * *p* < .05.

**Supplementary Table S6.** Results of the recurrent event regression analysis with laughter frequency (belly and fit of laughter) as the criterion with standardized predictors – **cyclical predictors**.

|  | Fixed | | | |
| --- | --- | --- | --- | --- |
|  | Coeff. | *B* | *SE* | *z* |
| Gender | β_1_ | 0.56 | 0.14 | 3.99*** |
| Age.cgm | β_2_ | -1.13 | 0.27 | -4.11*** |
| Time.hour.sin | β_3_ | 0.06 | 0.13 | 0.48 |
| Time.hour.cos | β_4_ | -0.41 | 0.08 | -5.35*** |
| Happiness.cgm | β_5_ | 0.24 | 0.02 | 10.75*** |
| Openness.cgm | β_6_ | 0.16 | 0.05 | 3.23** |
| Agreeableness.cgm | β_7_ | 0.01 | 0.05 | 0.13 |
| Conscientiousness.cgm | β_8_ | 0.18 | 0.05 | 3.32*** |
| Extraversion.cgm | β_9_ | 0.20 | 0.07 | 2.66** |
| Neuroticism.cgm | β_10_ | 0.04 | 0.13 | 0.31 |
| Gelotophobia.cgm | β_11_ | -0.08 | 0.08 | -0.94 |
| Life Satisfaction.cgm | β_12_ | 0.05 | 0.06 | 0.87 |
| STCI – bad mood.cgm | β_13_ | 0.24 | 0.13 | 1.91 |
| STCI – cheerfulness.cgm | β_14_ | 0.21 | 0.09 | 2.29* |
| STCI – seriousness.cgm | β_15_ | -0.18 | 0.08 | -2.42* |
| Gender:Age.cgm | β_16_ | 0.72 | 0.17 | 4.33*** |
| Gender:Time.hour.sin | β_17_ | -0.05 | 0.08 | -0.60 |
| Gender:Time.hour.cos | β_18_ | 0.26 | 0.05 | 5.15*** |

*Note*. *N* = 52, *n* recurrent events = 6,767, mean recurrent laughing events per subject = 130.1. Reference for gender was men. cgm = centered around the grand mean. *** *p* < .001, ** *p* < .01, * *p* < .05.
